# A Review of the Admission System for Mental Disorders in South Korea

**DOI:** 10.3390/ijerph17249159

**Published:** 2020-12-08

**Authors:** Dun-Sol Go, Kwon-Chul Shin, Jong-Woo Paik, Keun-A Kim, Seok-Jun Yoon

**Affiliations:** 1 Department of Health Care Policy Research, Korea Institute for Health and Social Affairs, Sejong 30147, Korea; kds237@gmail.com; 2 University of Seoul Law School, Seoul 02592, Korea; iskra0@daum.net; 3 The National Mental Health and Welfare Commission, Seoul 04933, Korea; paikjw@gmail.com; 4 Department of Psychiatry, School of Medicine, Kyung Hee University, Seoul 02453, Korea; 5 Department of Public Health, Graduate School, Korea University, Seoul 02841, Korea; keunak@korea.ac.kr; 6 Department of Preventive Medicine, Korea University College of Medicine, Seoul 02841, Korea

**Keywords:** mental health, suicide, mental health promotion and welfare act, involuntary admission, South Korea

## Abstract

This study presents a comprehensive overview of the characteristics of mental health problems and admission system in South Korea. We compared the mental health-related indicators data from South Korea to data from other Organization for Economic Cooperation and Development (OECD) countries. South Korea was identified as the country with the highest suicide rate, the longest length of stay in hospitals for mental disorders, and the highest number of psychiatric care beds. These results can be explained by considering the admission system for mental disorders. We reviewed the admission system and the Mental Health Promotion and Welfare Act, providing direction for improving the system.

## 1. Introduction

Among the Organization for Economic Cooperation and Development (OECD) members, South Korea has the highest number of psychiatric beds and the longest average length of hospitalization of psychiatric patients [1]. Although deinstitutionalization has led to more outpatient- and community-based care systems becoming important providers of treatment for patients with mental disorders, South Korea remains highly dependent on inpatient care. Differing characteristics of the state of mental health care in each country stem from the diversity of their systems and cultures. A nation’s healthcare system, including the payment system, affects the behavior of its providers and patients, and laws can affect the criteria for admission [2]. Additionally, resources, such as budgets and the availability of experts, determine psychiatric care characteristics. The number of mental health-related professionals per 100,000 persons in South Korea (30.6) was lower than the average for OECD countries (97.1) and varies by region from 45.1 to 14.6 [3].

In this study, we aimed to identify the characteristics of South Korea’s mental health treatment according to the OECD indicators and investigate issues related to the admission system. We analyzed data from the OECD health database extracted on 10 April 2020; all data refer to 2017 (or nearest year) [1]. We included all countries that provided data through the OECD health data system. The data on the mental health statistics of South Korea were obtained from the national mental health statistics published annually by the National Center for Mental Health from 2015 to 2019 [3,4,5,6,7] and from prior research [8].

## 2. Characteristics of South Korea’s Mental Health Problems

South Korea has shown consistent improvement in the overall health status, according to the OECD health data (see Table A1). Life expectancy at birth in South Korea was 82.7 years in 2017 (79.7 for men, 85.7 for women), while the average life expectancy among the OECD countries was 80.7 years (77.7 for men, 83.1 for women).

On the other hand, South Korea has had a relatively low rank in mental health status. South Korea had the highest suicide-related mortality rate among the member countries; mortality from suicide per 100,000 population was 24.6, while the overall average for the OECD was 11.5. Regarding perceived health status, the proportion of people over 15 years of age who perceived their health as “very good” or “good” was 29.5% in South Korea, the lowest among OECD countries; the OECD average was 68.0%.

The Global Burden of Disease Study (GBD) described “burden of disease” as a measure of “disability-adjusted life years” (DALYs), which is the sum of years of life lost (YLL) and years lived with disability (YLDs) [9,10]. The GBD Study reported 1693 YLDs attributable to MBDs per 100,000 persons in South Korea, lower than the OECD average of 1879 per 100,000 persons. However, YLLs attributable to MBDs in South Korea (1.8 per 100,000 persons) were higher than the OECD average (0.9 per 100,000 persons). These numbers imply that the burden of premature death from mental and behavioral disorders in South Korea was greater than average for OECD countries. In South Korea, MBDs were responsible for 7.5% of all DALYs (13.8% for YLDs, 0.017% for YLLs), whereas, among all OECD countries, the average burden of MBDs as a percentage of the total DALYs was 6.9% (13.9% for YLDs, 0.007% for YLLs). In addition, the higher age–sex-standardized ratio of excess mortality due to mental illness in South Korea than the averages for the 11 OECD countries indicate that patients diagnosed with schizophrenia or bipolar disorder were at greater risk of mortality compared to the general population in the countries that provided the excess mortality data.

The average length of stay for psychiatric patients in South Korea has remained high for years, and South Korea is the only country that has shown an increase in psychiatric beds. This shows that treatment for psychiatric patients in South Korea was concentrated in hospitalizations, while many other countries implemented policies to reduce the number of beds, turning support toward deinstitutionalized outpatient- and community-based care. These details are illustrated in Table 1.

## 3. Admission System for Mental Disorders

### 3.1. History of Mental Health Legislation in South Korea

The first Mental Health Act in South Korea was enacted in 1995 [11]. The Act categorized psychiatric admissions into four types: voluntary admission, involuntary admission by legal guardians, involuntary admission by administrative officials (Mayor, Governor, or Head of District), and emergency admission. To be admitted voluntarily, a patient can sign an application if they have received a diagnosis from a psychiatrist and can be discharged at any time at the patient’s request. Involuntary admission requires a diagnosis from one psychiatrist and consent of one legal guardian for a six-month hospitalization allowance. In 2008, the Act was revised, changing the required number of guardians for involuntary admission from one to two. Additionally, outpatient-based treatment was added as an option that may be recommended following a review of a patient’s admission extension.

Meanwhile, the United Nations Convention on the Rights of Persons with Disabilities 2014 committee report concerned the high involuntary admission rate and long-term hospitalization and recommended repealing the existing legal provisions allowing for the deprivation of liberty on the basis of disability, including psychosocial or intellectual disability [12]. The committee also suggested adopting measures to ensure that healthcare services, including all mental health care services, are based on the free and informed consent of the person concerned and to include a review system with the possibility of appeal.

In May 2016, the Mental Health Promotion and Welfare Act was submitted to the National Assembly by the government and has been enacted and implemented since 30 May 2017 [13]. The main purpose of the revised act was to address the problems associated with involuntary admissions, including reducing unnecessary admission and protecting patients’ rights by ensuring self-determination and welfare services during treatment. Under the Mental Health Promotion and Welfare Act, psychiatric admission was categorized into five types: voluntary admission, consented admission, involuntary admission by legal guardians, involuntary admission by administrative officials (Mayor, Governor, or Head of District), and emergency admission. Apart from the four types of psychiatric admissions, a new category for consented admission was established, allowing admissions that only require the consent of the patient and one legal guardian if the patient desires. The Act also strengthened the requirements for involuntary admission by requiring a diagnosis by two psychiatrists from different institutions for allowance of a three-month hospitalization period, whereas previously only one psychiatrist’s diagnosis was required for allowance of six-month hospitalization.

### 3.2. Psychiatric Beds in South Korea

After the implementation of the Mental Health Act, treatment for patients with mental illness was available in hospitals, resulting in an increasing number of public and private psychiatric beds. Table 2 shows the number of psychiatric beds from 1984 to 2017 [2,3,4,5,6,7]. The total number of psychiatric beds increased steadily and has decreased or maintained at a similar level since 2013. The proportion of beds in mental health hospitals increased, while the proportion of beds in mental nursing care facilities decreased; the psychiatric treatment environment was changed from facilities to hospitals.

### 3.3. Psychiatric Admissions in South Korea

Trends of psychiatric admissions in South Korea was shown in Table 3 [3,4,5,6,7]. In the early 1990s, the proportion of voluntary admissions was less than 10%, and that of involuntary admissions was more than 90% [14]. The proportion of voluntary admissions among total admissions continued to increase from 5.7% in 2000 to 35.6% in 2016, with an average annual growth rate of 14.6%. In particular, the proportion increased sharply between 2008 to 2010 compared to other years because the requirements of involuntary admission were strengthened by requiring two guardians instead of one.

Meanwhile, the proportion of involuntary admissions by family increased from 62.6% in 2000 to 74.0% in 2008 and decreased to 64.4% in 2016. The family-centered admission system limited the intervention of the investigation or review of the admission process by governmental institutions. In addition, the proportion of involuntary admissions by administrative officials decreased from 31.7% in 2000 to 7.6% in 2016. The patients who did not receive family care and those with mental disorders who were homeless were usually admitted by the administrative officials, including the Mayor, the Governor, or the Head of District. However, since the process of admission by the administration carries a high risk of violation of the human rights of patients, admission by administrative officials tended to decrease overall.

After the Mental Health Promotion and Welfare Act, as of the end of each year, the number of patients admitted voluntarily was 36,465 in 2017 and 35,577 in 2018. The new category, consented admission, had 12,325 patients admitted in 2017 and 15,115 in 2018. The number of patients admitted involuntarily was 28,371 in 2017 and 24,934 in 2018. The involuntary admissions in 2018 were the sum of admissions by legal guardians (21,045, 88.5%) and admissions by administrative officials (2746, 11.5%). After the Mental Health Promotion and Welfare Act was implemented, the proportion of voluntarily admitted patients, including consented admission, increased from 35.6% in 2016 to 63.2% in 2017 and 67.0% in 2018. On the other hand, the proportion of involuntarily admitted patients decreased from 64.3% in 2016 to 36.8% in 2017 and 33.0% in 2018.

### 3.4. Review and Decision on Admission for Mental Disorders

The admission review and decision is one of the most important processes in the deinstitutionalization of psychiatric treatment. One of the main changes made by the Mental Health Promotion and Welfare Act was the establishment of the independent review board for involuntary admission. The Committee for Examination as to Legitimacy of Admission was established at the five national mental hospitals. A total of 41,141 cases were reported for review from June 2018 to July 2019 after implementation of this system, with a monthly average of 3008 cases (excluding the first month). Under the Mental Health Act, there was no formal or national supervisory review procedure for six months after admission. The Mental Health Promotion and Welfare Act enabled a national early review stage at one month after admission. Furthermore, the information support system, developed for systemic management, enabled the implementation of a rapid process from reporting to notification. From July 2018 to June 2019, 573 cases (1.6%) out of 36,096 were discharged. The most frequent reason for the decision to discharge a patient was illegal coercive referral (physical limitations, assaults, compulsions), at 19.2%.

For the extension of admissions, the Mental Health Deliberative Committee, established under the Mental Health Act of 1995, reviewed the extension of psychiatric admissions by legal guardians or administrative officials after six months from the patient’s first admission. The 16 Metropolitan Mental Health Deliberative Committees under the control of the Mayor/Governor reviewed a total of 75,780 extensions of admissions in 2004, and the number of extensions increased to 75,945 in 2007 [15]. The discharge rate was 3.56%; however, the number of the committee’s actual decisions to discharge would be lower, as most of the patients were discharged prior to the review rather than due to the review committee’s determination.

After the revision of the Mental Health Act in 2008, the authority to review the extension of the admissions was transferred from the metropolitan Mayor/Governor to the Basic Mental Health Deliberative Committee under the control of the heads of administrative divisions including cities (*“Si”* in Korean), counties (*“Gun”* in Korean), and districts (*“Gu”* in Korean). A total of 145 Basic Mental Health Deliberative Committees reviewed a total of 73,353 extensions of admission in 2014, and 78,337 in 2017. The proportion of reviews decided to be discharged decreased from 3.9% in 2014 to 2.3% in 2017.

Through the Mental Health Promotion and Welfare Act, decision options have become diversified to include (i) Community treatment order, (ii) Re-review within three months, (iii) Conditional discharge, (iv) Transfer to another hospital, and (v) Conversion to voluntary admission. From June 2018 to June 2019, 361 cases (0.9%) out of 38,386 were decided to be discharged, 213 cases were decided to be reviewed in three months, and 16 community treatment orders were made.

## 4. Challenges Ahead for the Korean Mental Health System

South Korea has been making efforts to protect the rights of psychiatric patients and promote their rehabilitation and social restoration by diversifying admission types and establishing a review system. Several revisions of the legislation were aimed at shifting the focus to community-based mental health services but were insufficient to have a significant impact. We have outlined the limitations and challenges of the deinstitutionalization of the Korean mental health admission system.

### 4.1. Guarantee of Patient’s Opinion Statement

According to the United Nations’ (UN) Principles for the Protection of Persons with Mental Illness (MI), involuntarily admittance of patients includes procedural safeguards, and patients are guaranteed the right to submit evidence and face-to-face statements during the review process (MI 18-5) [16]. Since the implementation of the principles, both the court reviewers and the judges’ review agencies conduct face-to-face screening, which is a judicial procedure, and the right to submit evidence and face-to-face statements is guaranteed. Australia aims to guarantee patients’ right to self-determination by continuously reviewing the adequacy of inpatients through the Mental Health Tribunal, an independent quasi-judicial body. A mental health inquiry will be held at least two weeks after the involuntary admission and will provide an opportunity for patients to be questioned and to express their opinions directly or through a representative. The Mental Health Review Board must determine the admission period and give their judgement before the expiration of the period. In South Korea, when the head of a mental health hospital declares a notification of rights, they announce that the patient has the right to comment on the result of the review and provide a written form for this purpose, which can be used as material for review. However, only 23% of cases were reviewed face-to-face [8]. The person who meets the patient should be a member of the committee, not the interviewer. The committee should prepare various methods to provide the patients a chance to express their opinions, and they should listen to the patients’ opinions on the process of their admission, either face-to-face or through video meetings. In the case of video meetings, the number of reviewers can be decreased from five to three, and the number of cases reviewed can be increased.

### 4.2. Decision on Discharge and Implementation

Based on the Mental Health Promotion and Welfare Act, in addition to the decisions on admission and discharge, other decision options provided to patients and their families include (i) Community treatment order, (ii) Re-review within three months, (iii) Conditional discharge, (iv) Transfer to another hospital, and (v) Conversion to voluntary admission. However, even though the revised Act provides many more options compared to the original Mental Health Act, approximately 96% of the reviewed cases decisions were to extend admission [8]. Only 361 cases out of 38,386 (0.9%) were discharged, and 16 cases (0.04%) were given a community treatment order. The rate of discharge decisions through a review of the admissions was lower than that in other countries that have review systems. In Australia, the New South Wales’ Mental Health Tribunal reviewed a total of 6806 involuntary admission hearings for one year from July 2017 to June 2018; 15 cases (0.2%) were discharged, and 335 outpatient treatment orders (4.9%) were given [17]. On the other hand, the state of Victoria showed rates of 5% for discharge and 55% for community treatment orders. In Taiwan, the review committee reviewed 690 cases and decided to discharge 52 (7.5%). The main reason for the low rate of discharge decisions in South Korea is that the Act does not specify the subject and method of the community treatment order—that is, who should carry out and how. It is not easy to make a decision to discharge without being sure where and which treatment the patients will be offered after the discharge. When the committee reviews cases and made the community treatment orders, the care plan after the discharge should be reviewed and community centers and representatives to be transferred should be designated.

Since legislating the Mental Health Act, the Korean government has striven to lay the foundation of a community-centered system in the mental health sector over the last two decades. However, the re-admission rate to the hospital within one month after discharge was 37.9% [3]. Even if the patients transferred from hospital to community, the system to guarantee continuous treatment is insufficient.

Regional Mental Health Centers have played key roles in providing mental health services at the community level from treatment, counseling, rehabilitation, residential welfare services, and various social welfare services for patients and families. Insufficient community-based treatment resources lead to inadequate follow-up after discharge and early discontinuation of medication; this can lead to an increased suicide rate and chronic mental disorders. The Mental Health Promotion and Welfare Act, contrary to the old Mental Health Act, has a chapter on welfare services, such as education, employment, rehabilitation assistance, and cultural and sporting activities. However, compared to the scope of the work, the workforce and funding is insufficient. In 2019, there were 1839 mental health hospitals including 1299 out-patient clinics, 349 rehabilitation facilities and 315 community-based centers [7]. A total of 4425 patients used day hospital in 2019, and 43.8% of them used the outpatient facility in the hospital and 28.3% used outpatient clinics. A total of 87,075 patients registered community-based centers and rehabilitation facilities, and 30.5% of them used the services. Mental health workers per 100,000 population was 45.2, and 39.0% were professionals [7]. Most of the professionals (69.2%) worked at the hospital, and 17.4% worked at the community-based facilities. In addition, the budget for Mental Health Centers in community was made by matching funds from the central government and local government, the budget varies depending on the region. The per capita community mental health budget is 4791 KRW (4.35 USD) in 2018 and 5389 KRW (4.89 USD) in 2019 and differed more than three times depending on the region [7]. Differential support according to the number of populations and the size of the mental health problems should be provided. Unless additional investments in the infrastructure of mental health services are made as soon as possible, many communities may face difficulties in meeting the rising demand for community mental health services under the revised Act. According to countries experienced mental health reforms, including Italy, the United States, and the United Kingdom, the transformation of the mental health treatment and care from hospital-based setting to community-based mental health services has based on the various facilities. Nursing homes, community-based residential facilities, acute inpatient care facilities, day-hospitals, and centers have been established to provide care for mental disorders [18,19,20]. Community resources that can replace current hospital-based treatment should be diversified.

### 4.3. Measures for Other Related Systems

A designated person selected by the patient (a relative, friend, or colleague) is identified to receive notifications regarding the admission process. When a patient is admitted for a long period without their family’s care, the review committee asks the mental health centers in the community to develop a treatment plan with community support for the discharge of the patient. Moreover, per one of the UN Principles, decisions made through a hearing and the reasons for them must be prepared in writing, and a copy must be delivered to the patient or the patient’s agent and an attorney (MI 18-8). Patients or their guardians can request a trial for suspension of admission in family court, and a complaint procedure is provided through which they can appeal if they disagree with the trial proceedings or judgement.

The inpatient treatment system, including the functioning of the hospitals, needs to be divided into separate units—acute care, recovery, and long-term care—based on the inpatients ’characteristics [21]. The environment, and level or type of care, will depend on multiple factors: severity of the person’s mental condition, their physical health, and the type of treatment prescribed [22,23]. Providers and patients can prepare to return to the community, moving from acute care to recovery beds.

In addition to improving the physical environment, the psychological support system in hospitals needs to be enhanced. The national “peer support services” can be activated during inpatient treatment. The role of supporters includes providing information regarding the process of admission and treatment, assisting patients in expressing their opinions about the treatment, helping patients change their admission type, and providing a support system that continues after discharge [24].

The policies to change the paradigm of mental disorders treatment in South Korea, which is focused on inpatient treatment, should be activated and some are being attempted. Further studies on the effectiveness of these policies should be continued and used to establish strategies suitable for Korean system.

## 5. Conclusions

Among the nations examined, South Korea ranked as the country with the highest suicide rate, the longest length of stay in hospitals for mental disorders, and the highest number of psychiatric care beds. With the enactment of the Mental Health Promotion and Welfare Act, the proportion of involuntary admissions has decreased, and the mental health status of people in South Korea can be further improved by policies and systems that protect and guarantee patients’ rights. The Committee for Examination as to Legitimacy of Admission has been established as the national organization to review involuntary admissions, and admission decisions have been diversified. However, only 0.9% of the total cases reviewed were discharged. Through systematic improvement to the admission system, the policy and system should promote minimal hospital stays and support a return to daily life for psychiatric patients.

## Figures and Tables

**Table 1:** Comparison of mental health indicators for South Korea with the Organization for Economic Cooperation and Development (OECD) average.

Indicator		Value for South Korea and the Average for all OECD Countries ◼ South Korea ◼ OECD	South Korea’s Rank
Life expectancy (years)		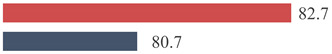	5
Perceived health status (%) (good or very good)		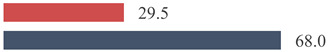	36
Suicide rates (per 100,000 persons)		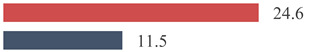	1
Burden of MBDs (per 100,000 persons)	YLLs	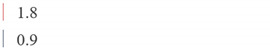	7
	YLDs	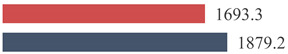	26
	DALYs	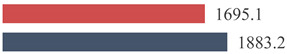	26
Excess mortality (age–sex-standardized ratio)	Schizophrenia	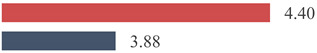	4
	Bipolar disorder	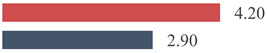	2
Psychiatric care beds (per 1000 population)		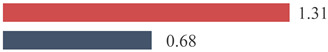	3
Average length of stay in hospitals (days)	Schizophrenia	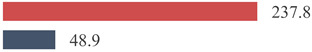	1
	Mood (affective) disorders	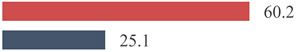	1

MBD: mental and behavioral disease; YLLs: years of life lost; YLDs: years lived with disability; DALYs: disability-adjusted life years.

**Table 2:** Number of psychiatric beds (per 100,000 persons) in South Korea.

Year	Total		Mental Health Hospitals			Mental Nursing Care Facilities		
	*N*	Per 100,000	*N*	Per 100,000	(%)	*N*	Per 100,000	(%)
1984	14,456	35.8	6107	15.1	42.2%	8349	20.7	57.8%
1990	31,541	73.5	14,109	32.9	44.7%	17,432	40.6	55.3%
1996	42,358	92.0	24,176	52.5	57.1%	18,182	39.5	42.9%
2000	58,010	122.0	43,885	92.3	75.7%	14,135	29.7	24.4%
2001	60,792	127.0	46,472	97.1	76.4%	13,960	29.2	23.0%
2002	63,708	132.4	49,868	103.6	78.3%	13,840	28.8	21.7%
2003	65,943	136.5	52,143	107.9	79.1%	13,886	28.7	21.1%
2004	67,241	138.7	53,391	110.1	79.4%	13,850	28.6	20.6%
2005	72,199	148.3	58,150	119.4	80.5%	14,049	28.9	19.5%
2006	78,056	159.7	63,760	130.4	81.7%	14,296	29.2	18.3%
2007	82,862	168.7	68,253	138.9	82.4%	14,609	29.7	17.6%
2008	83,937	169.9	69,702	141.1	83.0%	14,235	28.8	17.0%
2009	86,703	174.6	72,378	145.8	83.5%	14,325	28.8	16.5%
2010	89,559	179.5	75,414	151.2	84.2%	14,145	28.4	15.8%
2011	93,932	187.4	80,012	159.7	85.2%	13,920	27.8	14.8%
2012	98,428	195.5	84,220	167.3	85.6%	14,208	28.2	14.4%
2013	96,965	191.8	83,001	164.2	85.6%	13,964	27.6	14.4%
2014	97,515	192.1	83,711	164.9	85.8%	13,804	27.2	14.2%
2015	97,526	191.4	83,696	164.3	85.8%	13,830	27.1	14.2%
2016	96,924	189.6	83,405	163.2	86.1%	13,519	26.4	13.9%
2017	95,019	185.5	81,734	159.5	86.0%	13,285	25.9	14.0%
2018	92,422	180.2	79,257	154.5	85.8%	13,165	25.7	14.2%
2019	92,884	179.0	78,739	153.4	85.7%	13,145	25.6	14.3%

**Table 3:** Number of Psychiatric admissions by admission type in South Korea.

Year	Total	Voluntary Admission			Involuntary Admission					
		Voluntary	Consented	(%)	Legal Guardians		Others (Forensic)	Administrative Officials	Emergency	(%)
					Family	Mayor, Governor, or Head of District				
2000	59,032	3393	- ^*^	5.7%	36,945	18,694	-	-	-	94.3%
2001	60,079	4041	-	6.7%	39,167	16,868	-	-	-	93.3%
2002	61,066	3946	-	6.5%	40,263	16,857	-	-	-	93.5%
2003	64,083	4182	-	6.5%	41,853	17,293	755	-	-	93.5%
2004	65,349	5024	-	7.7%	44,024	15,618	683	-	-	92.3%
2005	67,895	6036	-	8.9%	45,958	15,316	585	-	-	91.1%
2006	70,967	6534	-	9.2%	49,935	13,917	579	-	-	90.8%
2007	70,516	6841	-	9.7%	51,028	11,961	686	-	-	90.3%
2008	68,110	9387	-	13.8%	50,425	7476	822	-	-	86.2%
2009	74,919	12,087	-	16.1%	50,575	11,154	851	176	76	83.9%
2010	75,282	15,271	-	20.3%	51,714	7027	910	251	109	79.7%
2011	78,637	16,833	-	21.4%	53,533	6853	1045	323	50	78.6%
2012	80,569	19,441	-	24.1%	53,105	6737	1013	230	43	75.9%
2013	80,462	21,294	-	26.5%	51,132	6320	1401	262	53	73.5%
2014	81,625	24,266	-	29.7%	49,792	6235	1159	147	26	70.3%
2015	81,105	26,064	-	32.1%	47,235	6432	1200	131	43	67.9%
2016	79,401	28,285	-	35.6%	43,643	6021	1300	94	58	64.4%
2017	77,161	36,465	12,325	63.2%	24,234	-	1570	2514	53	36.8%
2018	75,626	35,577	15,115	67.0%	21,045	-	1078	2746	65	33.0%

* The blank cells are years with no data, as the admission type had been discontinued or had not yet been created.

## Appendix A

**Table A1:** A. Mental health related indicators for OECD countries.

**Country**	**GDP per Capita**	**Life** **Expectancy** **at Birth**	**Perceived** **Health** **Status**	**Suicide** **Rates**	**Burden of Mental Disorders**						**Psychiatric** **Care Beds**	**Average Length of Stay in Hospitals**		**Excess Mortality (15–74 Years)**	
					**Rate of Burden of Disease**			**% of Total Burden of Disease**				**Schizophrenia** **Schizotypal and** **Delusional** **Disorders**	**Mood** **(Affective)** **Disorders**	**Schizophrenia**	**Bipolar Disorder**
					**YLD**	**YLL**	**DALY**	**YLD**	**YLL**	**DALY**					
**Unit**	**USD**	**Years**	**%**	**Per 100,000 Persons**	**Per 100,000 Persons**			**%**			**Per 1000 Persons**	**Days**		**Age–Sex Standardized Ratio**	
OECD	45,425	80.7	68.0	11.5	1879	0.9	1883	13.9	0.007	6.9	0.68	48.94	25.09	3.88	2.90
Korea	41,001	82.7	29.5	24.6	1693	1.8	1695	13.8	0.017	7.5	1.31	237.8	60.2	4.4	4.2
Rank of Korea	(20)	(5)	(36)	(1)	(26)	(7)	(26)	(24)	(4)	(14)	(3)	(1)	(1)	(4)	(2)
Australia	51,297	82.6	85.2	11.9	2317	0.77	2318	17.4	0.007	9.4	0.42	44.1	16.3		
Austria	54,652	81.7	71.7	12.4	1880	2.45	1883	13.7	0.018	6.9	0.61	34.7	21.9		
Belgium	50,772	81.6	74.4	15.9	1991	0.85	1992	14.1	0.006	7.0	1.36	9.9	14		
Canada	48,634	82.0	88.5	11.8	1938	0.73	1939	14.6	0.006	7.4	0.34	40.4	18.1		
Chile	23,657	80.2	59.7	10.7	2002	0.02	2002	16.5	0.000	8.1	0.10	64.9	18.1	3	1.3
Czech Republic	38,507	79.1	62	12.4	1522	0.16	1522	10.1	0.001	4.7	0.94	73.3	38.3		
Denmark	55,046	81.2	71.2	9.4	1850	0.50	1851	13.6	0.003	6.6	0.47	29.3	24.3	4.7	2.7
Estonia	33,867	78.2	52.5	13.0	1689	2.99	1692	11.6	0.015	4.9	0.53				
Finland	47,481	81.7	68.8	13.9	1970	1.08	1971	13.8	0.007	6.7	0.39	50.9	18.7	3.2	2.8
France	44,651	82.6	67.4	13.1	2142	1.18	2143	16.7	0.009	8.4	0.84	37.2	24.8		
Germany	53,012	81.1	65.4	10.2	2022	1.62	2023	14.1	0.010	6.5	1.28	35.2	35.9		
United Kingdom	45,988	81.3	74.8	7.3	1954	1.10	1955	14.1	0.008	7.0	0.38	99	44		
Greece	29,089	81.4	74	4.0	2089	0.79	2089	14.7	0.005	6.8	0.74	163	43		
Hungary	29,529	75.9	60.6	15.1	1578	0.16	1578	10.5	0.001	4.2	0.87	42.6	29.4		
Iceland	55,562	82.7	76.1	9.7	1843	0.19	1843	14.9	0.002	8.2	0.38	17.2	12.5		
Ireland	78,211	82.2	83.2	9.3	2047	0.21	2047	16.4	0.002	8.9	0.34	23.6	12.7		
Israel	38,983	82.6	74.1	5.4	1620	0.05	1620	14.7	0.001	8.4	0.41	64.1	29.3	3.8	3.7
Italy	41,785	83.0	77	5.7	1961	0.52	1962	14.0	0.004	7.2	0.09	15.9	15.8		
Japan	40,885	84.2	35.5	15.2	1668	2.02	1670	12.0	0.015	6.1	2.62				
Latvia	28,505	74.8	46.9	18.1	1603	0.15	1603	10.6	0.001	3.9	1.25	33.1	25.6	2	2.7
Lithuania	33,895	75.6	43.7	24.4	1764	0.02	1764	11.6	0.000	4.3	0.99	27.3	20.1	2	1.4
Luxembourg	112,702	82.2	71	7.2	1969	3.42	1972	14.3	0.030	7.8	0.76	49.4	21.9		
Mexico	20,023	75.0	65.5	5.4	1425	0.17	1425	14.9	0.001	5.6	0.03	45.9	14.1		
Netherlands	55,349	81.8	76.1	10.5	2151	2.18	2153	15.7	0.016	8.0	0.91	29.4	25	4	2.8
New Zealand	41,167	81.9	88.2	11.5	2217	0.44	2217	15.8	0.004	8.5	0.30	39.7	22	4.5	3.2
Norway	62,940	82.7	77.4	11.6	2078	0.77	2079	15.1	0.007	8.2	1.07	21.1	18.6	6.7	4.6
Poland	29,802	77.9	58.8	11.6	1420	0.63	1421	10.0	0.003	4.3	0.65	61.7	43.8		
Portugal	33,086	81.5	48.8	8.1	2102	0.01	2102	14.7	0.000	7.1	0.64	21.7	17.2		
Slovak Republic	30,911	77.3	67	9.7	1469	0.20	1584	10.5	0.001	4.6	0.81	36.2	26.6		
Slovenia	36,661	81.1	65.3	18.1	1584	0.15	1584	10.4	0.001	5.2	0.66	50.8	46.7		
Spain	39,627	83.4	74.2	6.8	2042	0.33	2042	15.6	0.003	8.1	0.36	56.6	24.3		
Sweden	52,693	82.5	76.5	11.1	2118	0.49	2118	15.6	0.004	8.1	0.43	48.8	18.8	4.4	2.5
Switzerland	67,139	83.6	80.2	11.2	1957	2.85	1959	14.2	0.028	8.1	0.93	34.5	31.4		
Turkey	28,209	78.1	68.8	2.6	1755	0.09	1755	15.1	0.001	7.4	0.05	14.5	13.2		
United States	59,984	78.6	87.9	13.9	2220	0.68	2220	15.4	0.004	7.1	0.21	10.1	6.4		
